# One-Pot Polymerization of Dopamine as an Additive to Enhance Permeability and Antifouling Properties of Polyethersulfone Membrane

**DOI:** 10.3390/polym12081807

**Published:** 2020-08-12

**Authors:** Sri Mulyati, Syawaliah Muchtar, Nasrul Arahman, Friska Meirisa, Yanna Syamsuddin, Zuhra Zuhra, Cut Meurah Rosnelly, Norazanita Shamsuddin, Normi Izati Mat Nawi, Mohd Dzul Hakim Wirzal, Muhammad Roil Bilad, Ryosuke Takagi, Hideto Matsuyama

**Affiliations:** 1 Department of Chemical Engineering, Universitas Syiah Kuala, Banda Aceh 23111, Indonesia; syawaliah2009@gmail.com (S.M.); nasrular@unsyiah.ac.id (N.A.); fmeirisa2005@gmail.com (F.M.); yanna_syamsuddin@unsyiah.ac.id (Y.S.); zuhra_74@yahoo.com (Z.Z.); cutnelly@ymail.com (C.M.R.); 2 Faculty of Integrated Technologies, Universiti Brunei Darussalam, Jalan Tungku Link BE1410, Brunei; norazanita.shamsudin@ubd.edu.bn; 3 Department of Chemical Engineering, Universiti Teknologi PETRONAS, Bandar Seri Iskandar 32610, Perak, Malaysia; normi_16000457@utp.edu.my (N.I.M.N.); mdzulhakim.wirzal@utp.edu.my (M.D.H.W.); mroil.bilad@utp.edu.my (M.R.B.); 4 Research Center for Membrane and Film Technology, Department of Chemical Science and Engineering, Kobe University, Rokkodai-Cho 1-1, Nadaku, Kobe 657-8501, Japan; takagi@harbor.kobe-u.ac.jp (R.T.); matuyama@kobe-u.ac.jp (H.M.)

**Keywords:** antifouling membrane, phase inversion, polydopamine, in-situ polymerization, peroxide-induced polymerization, additive blending

## Abstract

This paper reports the fabrication of polyethersulfone membranes via in situ hydrogen peroxide-assisted polymerization of dopamine. The dopamine and hydrogen peroxide were introduced into the dope solution where the polymerization occurred, resulting in a single-step additive formation during membrane fabrication. The effectivity of modification was evaluated through characterizations of the resulting membranes in terms of chemical functional groups, surface morphology, porosity, contact angle, mechanical strength and filtration of humic acid solution. The results confirm that the polydopamine was formed during the dope solution mixing through peroxide-assisted polymerization as proven by the appearance of peaks associated OH and NH groups in the resulting membranes. The presence of polydopamine residual in the membrane matric enhances the pore properties in terms of size and porosity (by a factor of 10), and by lowering the hydrophilicity (from 69° to 53°) which leads to enhanced filtration flux of up to 217 L/m^2^ h. The presence of the residual polydopamine also enhances membrane surface hydrophilicity which improve the antifouling properties as shown from the flux recovery ratio of > 80%.

## 1. Introduction

In recent years, membrane technology has been widely applied for purification processes in a variety of fields such as industries, wastewater treatment, etc., due to various merits, offering qualities such as its high efficiency of separation, ease of operation, and no secondary waste generation [1,2,3,4]. Common polymers such as polyvinylidene fluoride (PVDF), polysulfone (PSF) and polyethersulfone (PES) have been extensively used as materials for organic membrane production. However, the hydrophobicity nature of these polymers is maintained when applied as sole material for membrane fabrication and thus becomes a hindrance, causing strong adsorption of the foulant on the surfaces and pores of the resulting membranes [5,6,7]. Foulant adsorption is a consequential issue to the membrane as it leads to a decrease in water flux. Moreover, excessive use chemicals associated with membrane cleaning shortens the membrane life, which severely limits the development and practical application of membrane-based processes [8,9,10].

To deal with this problem, pursuit on the effective methods to reduce and control membrane fouling have been done through various endeavors [8,11,12,13]. Though the mechanism and behavior of fouling are complex, it has been widely reported that increasing the hydrophilicity of the membrane can be one of the viable solutions to limit the rate of foulant adsorption and thereby reducing the membrane fouling severity [14,15,16,17,18,19]. The enhancement of the hydrophilicity can be done by introducing a hydrophilic additive into the membrane materials through various techniques, such as blending [20,21,22,23,24], coating [25,26], grafting [27] and so forth.

Polydopamine is one of the chemicals that has been utilized effectively as an additive for development of hydrophilic membrane (by blending it with the main polymer in the dope solution) due to the plentiful presence of the polar hydroxyl groups [28,29,30]. The polar groups enhance the surface tension of the membrane surface when resides in/on the membrane matrix. Polydopamine itself is a product of dopamine polymerization through an oxidation reaction. The modification of the membrane commonly uses polydopamine instead of dopamine [31,32] as it contains more hydrophilic groups compared to its monomer and poses lower spherical hindrance to limit its leaching during membrane fabrication of the nonsolvent induced phase separation. Polydopamine also offers low mobility during the phase inversion as such leaches less in comparison to the dopamine [33].

Polydopamine is typically obtained from dopamine polymerization with the help of tris (tris hydroxymethyl aminomethane), ammonium persulfate, sodium periodate, or sodium chlorate, and copper sulfate buffer solution as oxidizing agents [34]. Though it has been reported that those chemicals are good oxidants for the polymerization of dopamine, only very few have been applied on site during membrane fabrication. In most cases, tris is the most frequently used oxidant to form polydopamine during membrane modification via coating [25], or combined blending–coating [34] methods. There were also reports on the uses of ammonium persulfate as an oxidant to trigger the formation of polydopamine which employed as an additive for modification of PVDF membrane through several novel techniques. They successfully obtained modified membranes with excellent water permeability and antifouling performances [33]. Besides those oxidants, it has also been reported that hydrogen peroxide (H_2_O_2_) is an excellent oxidizing agent for dopamine polymerization. It was used for polymerization of melanin for hair dye and recently used to trigger the growth of polydopamine for membrane coating [35,36,37] but not on-site during membrane fabrication.

This study investigates one-pot polymerization of dopamine into polydopamine using H_2_O_2_ as a polymerization agent in the dope solution prior used for membrane fabrication. The in situ polymerization was performed in a PES-based dope solution. After fabrication, the resulting membranes were assessed to envisage its effect on the enhancing membrane properties and hydraulic performance. The resulting membranes were then characterized in terms of chemical compositions, hydrophilicity, morphology, porosity and mechanical properties, as well as the filtration performances in terms of clean water permeability, and filtration of humic acid (HA) solution for evaluating the rejection and the membrane fouling resistance.

## 2. Materials and Methods

### 2.1. Materials

PES (Ultrason E6020 P, BASF, Ludwigshafen, Germany) and N-methyl-2-pyrrolidone (NMP, Merk, Kenilworth, NJ, USA) were used as the main polymer and solvent respectively for membrane fabrication. Dopamine in the form of 3-hydroxytyraminium chloride supplied from Merck (Kenilworth, NJ, USA) was used as an additive. It was polymerized into polydopamine with the help of H_2_O_2_ as an oxidizing agent. In addition, HA (Sigma Aldrich, St. Louis, MO, USA) was employed as a model contaminant for filtration experiment. Distilled water was used as a nonsolvent during immersion precipitation for membrane fabrication, solvent for the HA solution preparation and the feed for the pure water flux experiment.

### 2.2. Membrane Preparation

**Figure 1:**
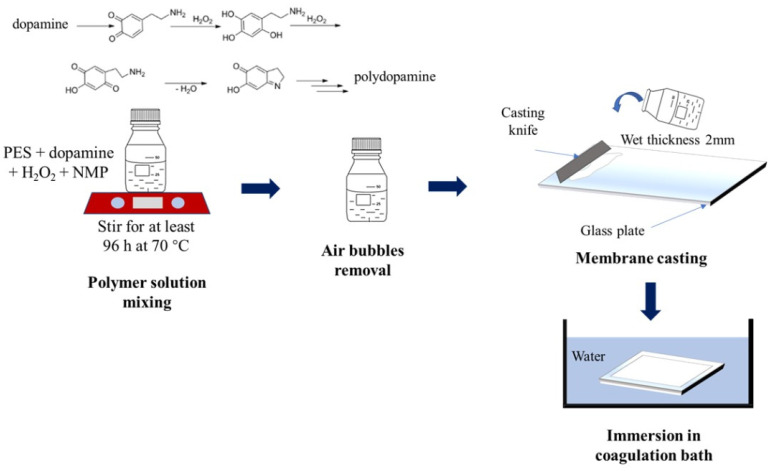
Illustration of membrane fabrication method showing the step of dophamine polymerization.

The fabrication of membrane was initiated by preparing the polymer solution composing of PES, dopamine, H_2_O_2_ and NMP. The detailed compositions of each component are given in Table 1. The mixture was stirred for 96 h at 70 °C vigorously until a homogeneous solution was formed. The homogeneous dope solution was then put under rest overnight in a fridge to evacuate entrapped air bubbles from stirring. The formation of homogeneous dope solutions suggest that dopamine is completely miscible with PES in the solvent. Nevertheless, no further characterization on the miscibility was performed. Following that, the dope solution was casted into a thin film by using a casting knifing with a wet thickness of 2 mm under room temperature. After casting, the cast film was immediately immersed in a water bath, also at atmospheric condition, to undergo nonsolvent-induced phase separation. The illustration of the membrane fabrication method is shown in Figure 1.

### 2.3. Membrane Characterization

To evaluate the effect of dope solution composition on the resulting membranes properties, several characterizations were performed. The Fourier-transform infrared spectroscopy (FTIR, Thermo Scientific iD5 ATR-Nicolet iS5 FTIR Spectrophotometer, Thermo Fisher Scientific, Waltham, MA, USA) was applied to distinguish the change in membrane chemical composition. Scanning electron microscopy (SEM, JSM-7500F, JEOL Ltd., Tokyo, Japan) was used to identify the membrane structure in terms of pore formation. To further study the pore characteristics, membrane porosity, which is defined as a fraction of voids in a membrane, was also measured by means of gravimetric technique, and the value was calculated using Equation (1).

(1) $$\varepsilon =\frac{\left(\omega_{w}-\omega_{d}\right)}{\rho \;As\;l}\times 100\%$$

where $\omega_{w}$ and $\omega_{d}$ represent the mass of the wet and dry membrane (kg), respectively. Meanwhile, ρ is the water density (998 kg/m^3^), As is the specimen surface area of the membrane sample (m^2^), and *l* represents the thickness (m) of the membrane sample.

The hydrophilicity of the membranes was evaluated through the measurement of contact angle of water droplet using Drop Master 300, Kyowa Interface Science Co., Nobitome Niiza, Japan by means of sessile drop technique. The measurement of membrane mechanical properties was also conducted according to the ASTM D638-14. Membrane specimen in a dumbbell (40 mm length; 4 mm width) form was stretched by Autograph (AGS-J, Shimadzu Co., Japan) tensile instrument at 20-N cell force until broken.

### 2.4. Filtration and Antifouling Experiments

The filtration experiment was conducted according to the procedure reported in our previous work [36] and was done using the same set-up. In brief, the filtration test was carried out in the following three stages: filtration of DI water to obtain pure water flux (*J_1_*) followed by filtration of 50 ppm HA solution to obtain the water flux of HA solution (*J_HA_*) immediately followed by backwashing using pure water. After that, the DI water was filtrated again through the same membrane sample to obtain the second pure water flux (*J_2_*). The whole procedure was conducted at a feed pressure of 1 barg, meanwhile, the backwash procedure was carried out for 20 min at 0.1 barg. The permeate was collected and weighted at interval of 10 min for flux determination, and the flux was calculated using Equation (2). As for the filtration of HA solution, the permeate collected and the HA concentration was analyzed by UV-VIS spectrophotometry (Spectrometer UV-Vis 1800, Shimadzu, Japan). The HA rejection was calculated using Equation (3).

(2) $$J=\frac{V}{A\Delta t}$$

(3) $$R=\left(1-\frac{C_{p}}{C_{f}}\right)\times 100\%$$

where *J* is flux (L/(m^2^.h), *V* volume of permeate (L), *A* membrane area (m^2^), Δ*t* permeate collection time (h), *R* rejection (%), *C_p_* concentration of the HA in the permeate (ppm) and *C_f_* concentration of the HA in the feed (ppm).

The antifouling properties of the membranes were evaluated in terms of recovery ratio of pure water flux (*FRw*), the total flux loss due to fouling (*R_t_*), the recoverable flux loss (*R_r_*), and loss of flux that cannot be recovered (*R_ir_*) by using Equations (4)–(7):

(4) $$FRw=\left(\frac{J_{2}}{J_{1}}\right)$$

(5) $$R_{t}=\left(\frac{J_{2}-J_{HA}}{J_{1}}\right)$$

(6) $$R_{r}=\left(\frac{J_{2}-J_{HA}}{J_{1}}\right)$$

(7) $$R_{ir}=\left(\frac{J_{1}-J_{2}}{J_{1}}\right)$$

## 3. Results and Discussions

### 3.1. Membrane Chemical Composition

**Figure 2:**
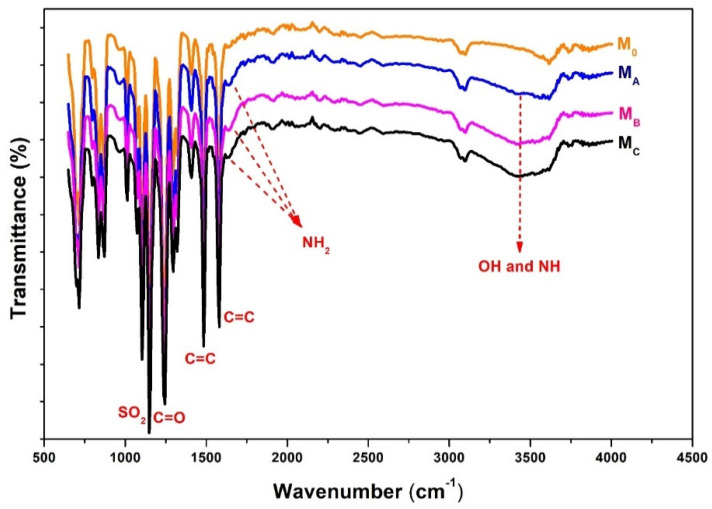
Infrared spectra of pure PES membranes without and with addition of dopamine in the dope solution.

The polydopamine was formed and was found still present in the membrane matrix as shown from the results of the FTIR in Figure 2. Changes in the chemical composition of the PES membranes with and without polydopamine additive can be seen by comparing the FTIR peaks. It shows a quite striking difference of the spectra of the membrane sample made from pure PES (M_O_) and the ones blended with polydopamine (M_A_, M_B_, M_C_). The difference is marked by the appearance of new transmission peaks at a wavenumber of 1640 cm^−1^ and in the wavenumber range of 3000–3600 cm^−1^. These peaks indicate the presence of NH and OH bonds in the polydopamine which is a compound composed of long and repeated catechol chains (NHOH) [30,38], present in polydopamine. Both of these groups have a high affinity to water which causes polydopamine to be highly hydrophilic. From the results, it can be concluded that the polydopamine was formed through polymerization of dopamine during the preparation of the dope solution. The residual polydopamine in the membrane matrix is detected from the FTIR spectra as shown in Figure 2.

The IR measurement was performed on the top surface side of the membrane samples. As shown in Figure 2, there is only slight difference between the pristine and modified membranes. We assume it could be because the membranes were not completely dried when the analysis was performed hence the spectra results are somewhat identical especially at the wavenumber range of 3000–3600 cm^−1^ which is the reflection of -OH groups. However, with addition of polydopamine, the transmission peak in that area becomes broader and higher in intensity with increasing additive concentration. Moreover, at a wavenumber of 1640 cm^−1^, there is a new peak representing NH_2_ vibration emerging on modified membranes which cannot be seen in the pure membrane.

**Figure 3:**
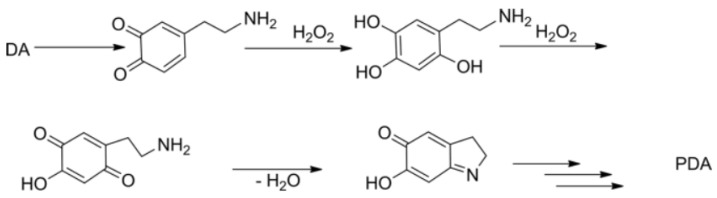
Oxidation of dopamine to polydopamine by hydrogen peroxide.

The oxidative self-polymerization of dopamine is a complicated process, as illustrated in Figure 3. This process occurs through covalent cross-linking and non-covalent physical assembly. In this research, the polymerization process of dopamine to polydopamine occurred during the preparation of the membrane casting solution with the presence of hydrogen peroxide as an oxidizer. The mechanism of the peroxide-assisted formation of dopamine into polydopamine occurs by involving redox reactions. As reported by Manini [39], that the pathway of dopamine polymerization induced by peroxide is initiated by the transformation of dopamine to dopaminequinone followed by the formation of 6-hydroxydopamine quinone and then to 5,6-dihydroxylindole marked by the change in color of the solution from clear to brown which was observed during the dope solution mixing. After several advanced stages, polydopamine particles are formed as also suggested elsewhere [40].

### 3.2. Surface Morphology and Surface Pore Size

**Figure 4:**
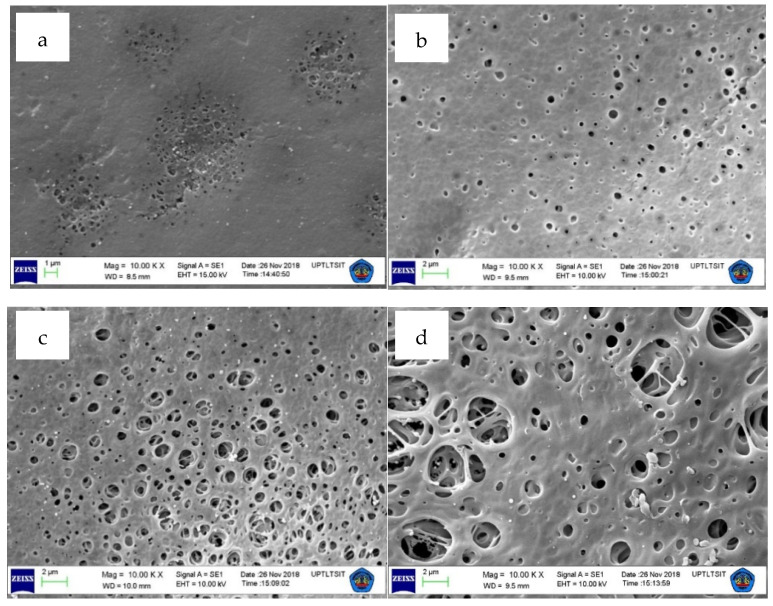
Surface morphological of (**a**) M0, (**b**) MA, (**c**) MB, and (**d**) MC membranes.

The effects of modification on the surface morphology of the PES membranes obtained using SEM are presented in Figure 4. It shows that the surface morphologies of the PES membranes exhibit significant changes with the addition and the amount of dopamine loadings. These changes can be seen from the shape, size, and number of pores formed on the surface of the membrane. The M_0_ (non-modified) membrane has a surface with less in pore number, smaller in pore sizes and uneven in-surface pore distribution compared to those on the surfaces of M_A_, M_B_, and M_C_ membranes. Such change can be attributed to the role of polydopamine as the pore-forming agents, as also reported elsewhere [33,36]. The presence of residual polydopamine possibly due to intertwines with the PES chains, and the role of polydopamine as “pore forming agent” is also proved from membrane morphology data in Figure 4 and the porosity data discussed later.

Hydrophilic polydopamine molecules have a high affinity to water. Therefore, during the immersion–precipitation process, the polydopamine particles contained in the membrane tend to be attracted to the membrane surface (water-reach phase) or even leached out into the water (bulk or polymer-lean phase). The particles detached from the membrane system leave marks on the surface of the membrane film in the form of pores [36,41]. Figure 4 also shows that the concentration of the dopamine influences the surface morphology of the membrane in which the higher dosing of the dopamine in the casting solution, the more abundant and the larger the pores formed.

The findings on the role of polydopamine in enhancing surface pore properties is in line with the one reported earlier. Jiang et al. [33] reported three different methods of polydopamine incorporation into the PVDF membrane by using ammonium persulphate (APS) as an oxidizing agent. They compared the in situ polymerization (M1), by dissolving dopamine and APS in water-DMAc solution followed by addition of PVDF (M2), and lastly the polydopamine was separately prepared then used as the additive (M3). According to their results, the first method resulted in better performing membrane than the rests (M1 > M2 > M3). Though in terms of polymerization, M1 had a slower rate of polymerization due to existence of DMAc (poor solvent for dopamine), but during membrane formation, M1 had outstanding surface pore and connectivity. The M3 had less unpolymerized dopamine residue due to high rate polymerization. However, its addition to the membrane system only managed to create a large in number and size of inner macro pores during membrane formation but not the surface pores and their connectivity. This finding was used as basis for designing the experiment in this study, which focused on the exploration of in situ polymerization during the preparation of the dope solution for membrane fabrication.

### 3.3. Porosity

**Figure 5:**
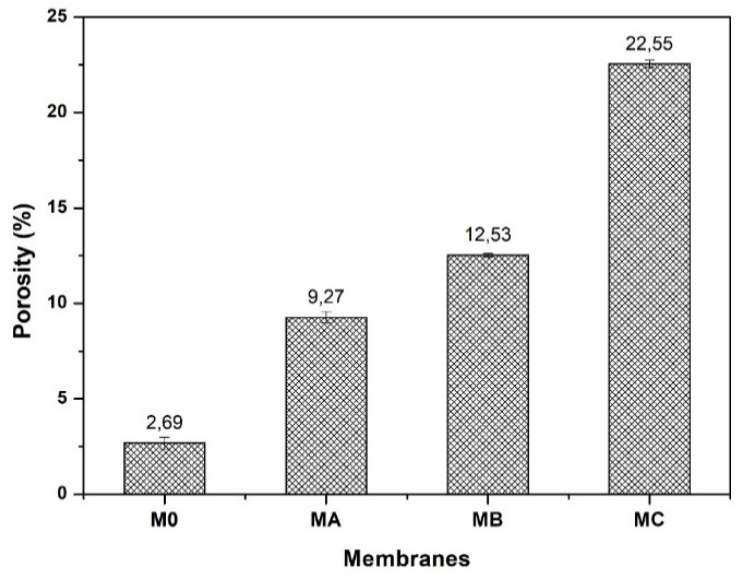
Effects of dopamine addition on membrane porosity.

The porosity data of all the membrane samples is shown in Figure 5. In general, the porosity of the modified membranes is much higher than that of pure PES membrane (M_0_). The porosity of pure PES (M_0_) is only 2.69%. With the addition and increasing concentration of dopamine, the porosity increases sharply up to 10 times. An increase in the dopamine loading from 1% to 3% enhances the porosity of the resulting membranes from 9.27% to 22.55%. The enhancement of porosity was caused by the presence of the polydopamine in the dope solution [42]. At a higher dopamine loading, more and larger polydopamine particle/clusters were formed hence the pore properties of the membrane also increased. This finding is well supported by the SEM analysis results shown in Figure 4.

### 3.4. Hydrophilicity

**Figure 6:**
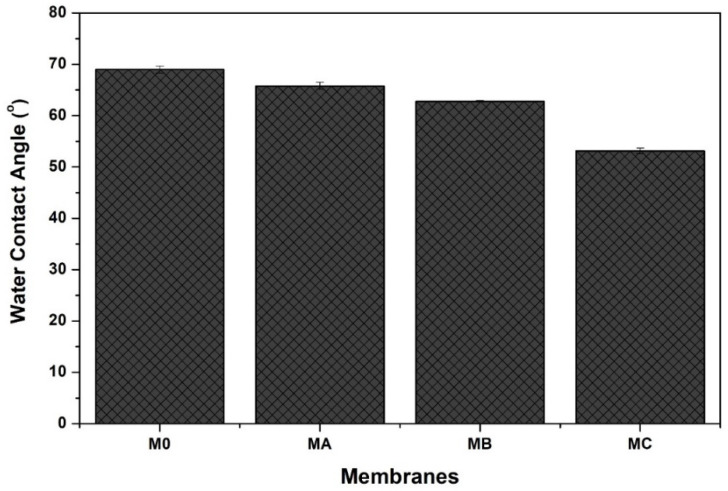
Water contact angle of pure PES and blended PES membrane with polydopamine of different concentrations.

The effect of polydopamine loading on the resulting membrane is shown in Figure 6. Since polydopamine is well known for its hydrophilic properties, one can expect its active role in enhancing the membrane hydrophilicity when it resides near by the membrane surface, as demonstrated in Figure 2. Figure 6 also shows that all the polydopamine-blended membranes have higher hydrophilicity (ascribed by the smaller contact angle) than the pure PES membrane (M_0_). The increase in hydrophilicity trend is in line with the dopamine loadings. The improvement of membrane hydrophilicity with the addition of dopamine is directly related to the presence of abundance hydrophilic catechol moieties from polydopamine as well as the porous morphological surface of blended membranes as proven by the FTIR and SEM results in Figure 2 and Figure 4, respectively.

### 3.5. Membrane Mechanical Properties

**Figure 7:**
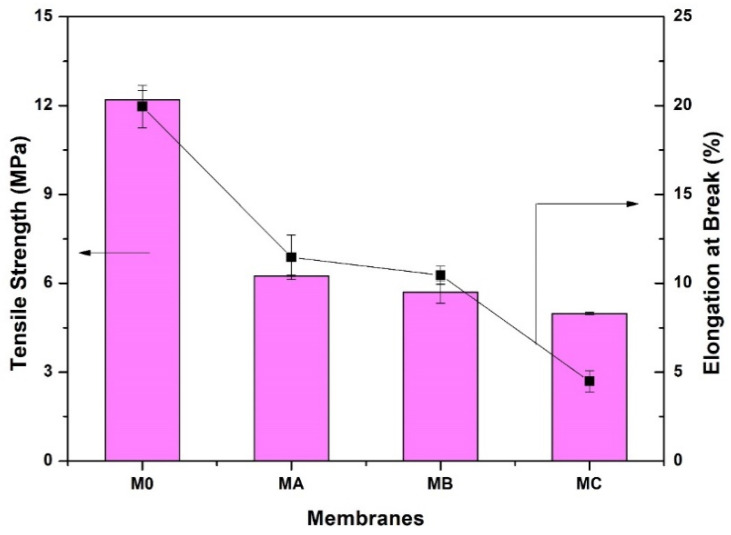
Effects of dopamine addition on mechanical properties of the membranes.

Figure 7 shows that the presence of polydopamine in the membrane system also affects the mechanical properties of the fabricated membranes, especially at high dopamine concentrations. The mechanical properties of a membrane must be considered as it greatly affects its durability under high hydraulic pressure. This shows that morphological parameters such as pore size and structure have an influence on the polymer membrane properties because, if the pore size increases, the mechanical strength decreases [43]. As shown in Figure 7, the pure PES membrane has excellent mechanical traits with 12 MPa of tensile strength with 20% elongation at the break. The addition of 1% dopamine into the dope solution (membrane M_A_) resulted in a decline of mechanical strength to 6.25 MPa for tensile and 11% elongation at break. A further increase in dopamine loading leads to severe deterioration of both tensile strength and elongation at the break. This behavior is closely related to the morphology of the membranes. The addition of dopamine resulting in a membrane with more porous structure which causes the membrane to be less ductile and easily broken when placed under load/pressure. This is also because the membrane retains water which reduces the mechanical stability of the membrane.

### 3.6. Hydraulic Performance

**Figure 8:**
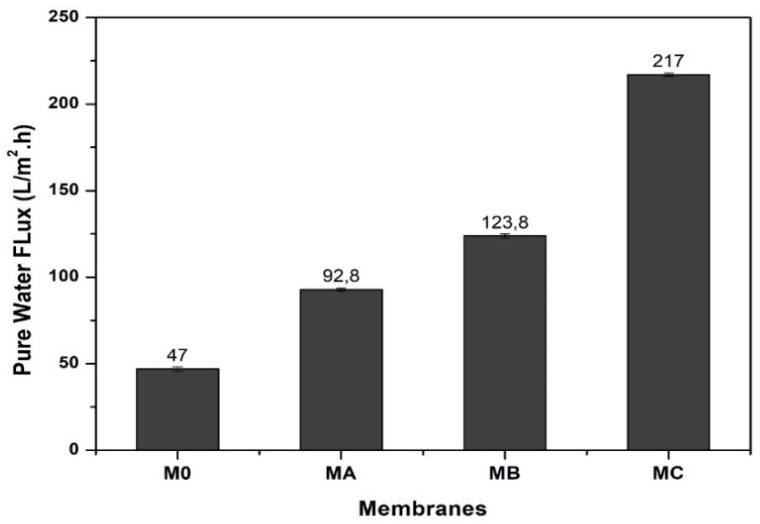
Effects of dopamine loading on the pure water flux.

The pure water flux for pure PES and dopamine-modified membranes are shown in Figure 8. For pure PES membranes (M_A_), the generated pure water flux is only 47.06 L/m^2^.h. Meanwhile, after the addition of dopamine, the pure water flux improves significantly to 92.75; 123.80; and 216.99 L/m^2^.h for dopamine loadings of 1%, 2% and 3%, respectively. The increase in the values of the membrane pure water fluxes is due to an increase in pores (number and size) as well as an increase in the hydrophilic nature of the membrane as shown in Figure 3 and Figure 6, respectively.

**Figure 9:**
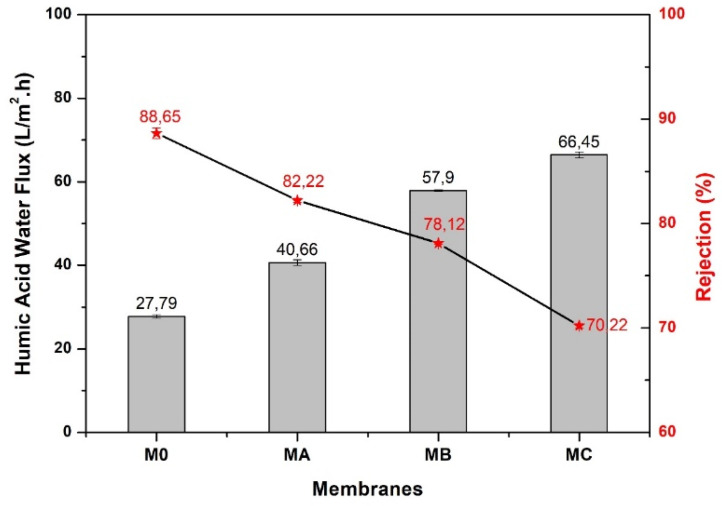
Water permeability and rejection of HA solution.

The same flux trend is also seen in the flux performance of filtration using the HA solution as a model fouling feed, as shown in Figure 9. The addition of dopamine as additive also affects the selectivity of the resulting membranes as shown by the rejection of HA (Figure 9). In general, for all membranes, the rejection values are inversely proportional to the water flux. This is caused by the pore properties of the membranes. The high pore number and large pore size facilitate water to pass through the membrane thus the amount of generated permeate increases. However, large pores (especially with a larger size) result in more HA passing along into the permeate, hence decreases the rejection. This phenomenon is seen in all membranes with dopamine additive especially those with higher loadings (M_B_ and M_C_). In contrast, pure PES membrane poses the highest rejection rate of 88.65% because the PES membrane has surface pores with denser structure, smaller in size and fewer in numbers (refers to Figure 4). This surface characteristic of pristine PES causes not only largely retaining the HA particles, but also restricting the flow of water leading to a low water flux.

### 3.7. Fouling Endurance Test

For all membrane-based filtration processes, membrane fouling, i.e., deposition of solute materials on or into the membrane that leads to a decrease in flux performance due to increasing resistance. Cake formation, suspension flow properties, and fouling layer properties have been widely reviewed for microfiltration and ultrafiltration, and it has been recognized that membrane performance severely decreases unless the membrane fouling is adequately managed [8,44,45,46].

**Figure 10:**
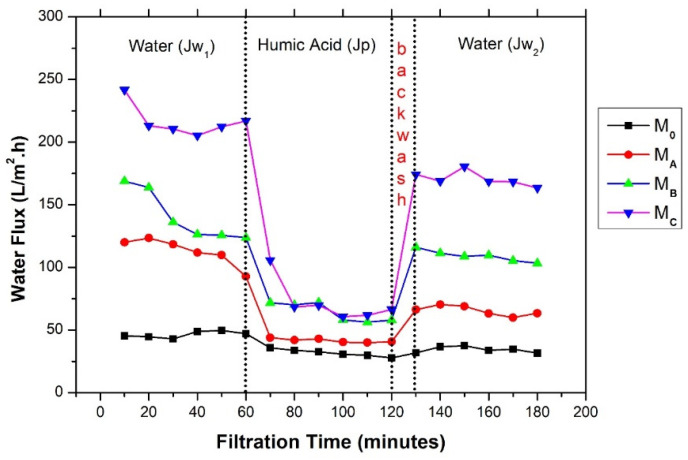
The profile of water flux decline and the recovery during filtration of DI water and HA solution.

The membrane fouling can be observed from the obvious decrease in water flux during the filtration process. Figure 10 shows the flux performance of the prepared membranes in one round of a three-stage filtration experiment. The flux recovery ratio is defined as the ratio of pure water flux obtained before and after the backwash process [33]. It appears that initially, all membranes posed high pure water flux (*Jw_1_*) which decreased when the clean water feed was replaced by the HA solution. This decrease is caused by the membrane fouling due to the accumulation of HA particles on the membrane surface. To remove the accumulated HA foulant and to restore the clean water flux, membrane cleaning is necessary. The effectiveness of cleaning can be determined based on the second water flux profile (*Jw_2_*). The closer the value of *Jw_2_* to *Jw_1_*, the easier is the membrane cleaning, which reflects better antifouling properties.

**Figure 11:**
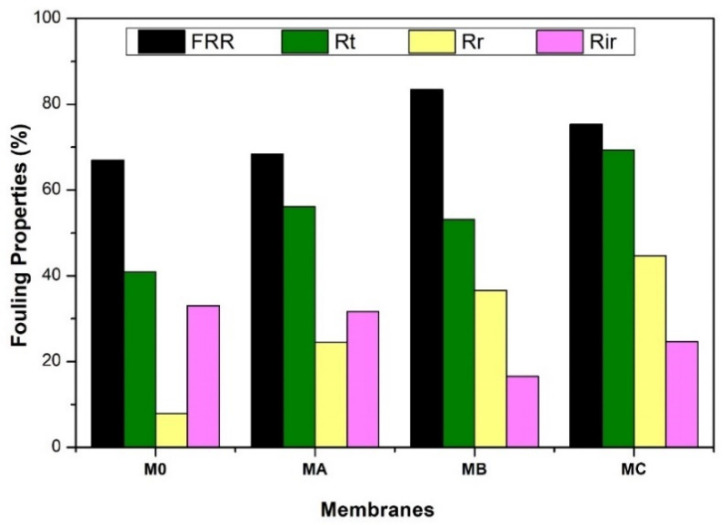
Antifouling performance of PES membranes without and with modification using dopamine at different concentrations.

Figure 11 shows the antifouling performance of all prepared membranes. In general, it shows that the dopamine-blended membranes pose better antifouling properties than the pure PES membrane in terms of *FRw, R_t_, R_r_* and *R_ir_*. The M_A_ and M_B_ have higher *FRw* and lower *R_t_* compared to the M_0_ after the membrane cleaning. The trend can be explained as follows. The M_0_ has relatively smaller pore sizes as confirmed by the SEM images and high rejection. The dense surface characteristic causes HA to accumulate easily and rapidly on the membrane material causing a total flux loss (*R_t_*) of 40.94%. Even after backwashing, only 7.89% of the initial water flux was recovered, most likely due to the strong hydrophobic–hydrophobic interaction between the surface of the M_0_ and HA molecules [36,47].

Interestingly, despite the claim of a better antifouling property, membranes containing polydopamine have higher total flux loss than that of the plain PES membrane. The total flux lost reaches 69% for the membrane with a dopamine concentration of 3% (M_C_). This is due to the morphology of the polydopamine-containing membranes. The presence of more pores on the membrane surface provides more contact areas for the HA molecules to adhere which results in greater fouling [36]. However, the presence of polydopamine increases the hydrophilicity of the membrane leading to better removal of foulant through simple cleaning as observed from the Rr and Rir data in Figure 11. For example, the fouling in M_A_ caused as much as 18.92% flux loss from the initial water flux. After cleaning, 13.50% of the flux loss was recovered, corresponding to *FRw* of up to 71.35%.

## 4. Conclusions

Enhanced properties and hydraulic performance of PES-based membranes were achieved through peroxide-assisted dopamine in situ polymerization in the dope solution. The FTIR results confirm the existence of the dopamine polymerization reaction assisted by peroxide. The existence of residual polydopamine in the membranes was proven by the appearance of OH and NH groups. The presence of polydopamine as additive enhanced the pore properties in terms of size and porosity as demonstrated by the SEM imaging results. Polydopamine increases membrane hydrophilicity, thus enhancing the permeability and fouling reversibility. In addition, due to the improved pore characteristic, the hydraulic performance reaches a flux of 200 L/m^2^.h. The increased hydrophilicity brought about the excellent antifouling properties of the membrane with an FRR of pure water flux of up over 80% (for M_B_). The overall results suggest that a straightforward method of incorporating polydopamine as an additive for PES membrane fabrication could be done by an efficient one-pot polymerization method. The resulting membranes also pose enhanced antifouling and substantially higher operational flux which are attractive to be applied for filtration of fouling prone feeds.

## Figures and Tables

**Table 1:** Compositions of dope solution for membrane preparation.

Membrane ID	PES (wt %)	Additive (wt %)		Solvent (wt %)
		Dopamine	H_2_O_2_	NMP
M_O_	15	-	-	85
M_A_	13.5	1	0.5	85
M_B_	12	2	1	85
M_C_	10.5	3	1.5	85

## References

[1] Saleem H., Trabzon L., Kilic A., Zaidi S.J. (2020). Recent advances in nanofibrous membranes: Production and applications in water treatment and desalination. Desalination.

[2] Zuo H.-R., Shi P., Duan M. (2020). A review on thermally stable membranes for water treatment: Material, fabrication, and application. Sep. Purif. Technol..

[3] Nabeel F., Rasheed T., Bilal M., Iqbal H.M. (2019). Supramolecular membranes: A robust platform to develop separation strategies towards water-based applications. Sep. Purif. Technol..

[4] Ibrahim N., Wirzal M., Nordin N., Abd Halim N. Development of Polyvinylidene fluoride (PVDF)-ZIF-8 Membrane for Wastewater Treatment. Proceedings of the 4th International Conference on Civil and Environmental Engineering for Sustainability (IConCEES 2017), IOP Conference Series: Earth and Environmental Science.

[5] Zeng K., Zhou J., Cui Z., Zhou Y., Shi C., Wang X., Zhou L., Ding X., Wang Z., Drioli E. (2018). Insight into fouling behavior of poly (vinylidene fluoride)(PVDF) hollow fiber membranes caused by dextran with different pore size distributions. Chin. J. Chem. Eng..

[6] Shoparwe N.F., Otitoju T.A., Ahmad A.L. (2018). Fouling evaluation of polyethersulfone (PES)/sulfonated cation exchange resin (SCER) membrane for BSA separation. J. Appl. Polym. Sci..

[7] Sakinah A.M., Ismail A., Illias R.M., Hassan O. (2007). Fouling characteristics and autopsy of a PES ultrafiltration membrane in cyclodextrins separation. Desalination.

[8] Shi X., Tal G., Hankins N.P., Gitis V. (2014). Fouling and cleaning of ultrafiltration membranes: A review. J. Water Process Eng..

[9] Abd Halim N.S., Wirzal M.D.H., Bilad M.R., Md Nordin N.A.H., Adi Putra Z., Sambudi N.S., Mohd Yusoff A.R. (2019). Improving Performance of Electrospun Nylon 6, 6 Nanofiber Membrane for Produced Water Filtration via Solvent Vapor Treatment. Polymers.

[10] Abd Halim N.S., Wirzal M.D.H., Bilad M.R., Md Nordin N.A.H., Adi Putra Z., Mohd Yusoff A.R., Narkkun T., Faungnawakij K. (2019). Electrospun Nylon 6, 6/ZIF-8 Nanofiber Membrane for Produced Water Filtration. Water.

[11] Goh P., Lau W., Othman M., Ismail A. (2018). Membrane fouling in desalination and its mitigation strategies. Desalination.

[12] Bagheri M., Akbari A., Mirbagheri S.A. (2019). Advanced control of membrane fouling in filtration systems using artificial intelligence and machine learning techniques: A critical review. Process Saf. Environ. Prot..

[13] Mulyati S., Muchtar S., Yusuf M., Arahman N., Sofyana S., Rosnelly C.M., Fathanah U., Takagi R., Matsuyama H., Shamsuddin N. (2020). Production of High Flux Poly (Ether Sulfone) Membrane Using Silica Additive Extracted from Natural Resource. Membranes.

[14] Younas H., Zhou Y., Li X., Li X., Sun Q., Cui Z., Wang Z. (2019). Fabrication of high flux and fouling resistant membrane: A unique hydrophilic blend of polyvinylidene fluoride/polyethylene glycol/polymethyl methacrylate. Polymer.

[15] Mu K., Zhang D., Shao Z., Qin D., Wang Y., Wang S. (2017). Enhanced permeability and antifouling performance of cellulose acetate ultrafiltration membrane assisted by L-DOPA functionalized halloysite nanotubes. Carbohydr. Polym..

[16] Zhao X., Zhang R., Liu Y., He M., Su Y., Gao C., Jiang Z. (2018). Antifouling membrane surface construction: Chemistry plays a critical role. J. Membr. Sci..

[17] Halim N., Wirzal M., Bilad M., Yusoff A., Nordin N., Putra Z., Jaafar J. Effect of solvent vapor treatment on electrospun nylon 6, 6 nanofiber membrane. Proceedings of the International Conference on Advanced Manufacturing and Industry Applications, IOP Conference Series: Materials Science and Engineering.

[18] Mat Nawi N.I., Abd Halim N.S., Lee L.C., Wirzal M.D.H., Bilad M.R., Nordin N.A.H., Putra Z.A. (2020). Improved nylon 6, 6 nanofiber membrane in a tilted panel filtration system for fouling control in microalgae harvesting. Polymers.

[19] Arahman N., Maimun T., Bilad M. (2018). Fabrication of polyethersulfone membranes using nanocarbon as additive. Int. J. Geomate.

[20] Wahab M.Y., Muchtar S., Jeon S., Fang L.F., Rajabzadeh S., Takagi R., Arahman N., Mulyati S., Riza M., Matsuyama H. (2019). Synergistic effects of organic and inorganic additives in preparation of composite poly (vinylidene fluoride) antifouling ultrafiltration membranes. J. Appl. Polym. Sci..

[21] Al-Husaini I.S., Yusoff A.R.M., Lau W.-J., Ismail A.F., Al-Abri M.Z., Wirzal M.D.H. (2019). Iron oxide nanoparticles incorporated polyethersulfone electrospun nanofibrous membranes for effective oil removal. Chem. Eng. Res. Des..

[22] Al-Husaini I., Yusoff A., Lau W., Ismail A., Al-Abri M., Al-Ghafri B., Wirzal M. (2019). Fabrication of polyethersulfone electrospun nanofibrous membranes incorporated with hydrous manganese dioxide for enhanced ultrafiltration of oily solution. Sep. Purif. Technol..

[23] Marbelia L., Bilad M.R., Vankelecom I.F. (2019). Gradual PVP leaching from PVDF/PVP blend membranes and its effects on membrane fouling in membrane bioreactors. Sep. Purif. Technol..

[24] Arahman N., Mulyati S., Fahrina A., Muchtar S., Yusuf M., Takagi R., Matsuyama H., Nordin N.A.H., Bilad M.R. (2019). Improving Water Permeability of Hydrophilic PVDF Membrane Prepared via Blending with Organic and Inorganic Additives for Humic Acid Separation. Molecules.

[25] Muchtar S., Wahab M.Y., Fang L.F., Jeon S., Rajabzadeh S., Takagi R., Mulyati S., Arahman N., Riza M., Matsuyama H. (2019). Polydopamine-coated poly (vinylidene fluoride) membranes with high ultraviolet resistance and antifouling properties for a photocatalytic membrane reactor. J. Appl. Polym. Sci..

[26] Foong C.Y., Wirzal M.D.H., Bustam M.A. (2020). A review on nanofibers membrane with amino-based ionic liquid for heavy metal removal. J. Mol. Liq..

[27] Vatanpour V., Zoqi N. (2017). Surface modification of commercial seawater reverse osmosis membranes by grafting of hydrophilic monomer blended with carboxylated multiwalled carbon nanotubes. Appl. Surf. Sci..

[28] Yang H.-C., Luo J., Lv Y., Shen P., Xu Z.-K. (2015). Surface engineering of polymer membranes via mussel-inspired chemistry. J. Membr. Sci..

[29] Li B., Liu W., Jiang Z., Dong X., Wang B., Zhong Y. (2009). Ultrathin and stable active layer of dense composite membrane enabled by poly (dopamine). Langmuir.

[30] Liebscher J.R., Mrówczyński R., Scheidt H.A., Filip C., Hadade N.D., Turcu R., Bende A., Beck S. (2013). Structure of polydopamine: A never-ending story?. Langmuir.

[31] Huang Q., Chen J., Liu M., Huang H., Zhang X., Wei Y. (2020). Polydopamine-based functional materials and their applications in energy, environmental, and catalytic fields: State-of-the-art review. Chem. Eng. J..

[32] Wang Z., Yang H.-C., He F., Peng S., Li Y., Shao L., Darling S.B. (2019). Mussel-inspired surface engineering for water-remediation materials. Matter.

[33] Jiang J.-H., Zhu L.-P., Zhang H.-T., Zhu B.-K., Xu Y.-Y. (2014). Improved hydrodynamic permeability and antifouling properties of poly (vinylidene fluoride) membranes using polydopamine nanoparticles as additives. J. Membr. Sci..

[34] Wei Q., Zhang F., Li J., Li B., Zhao C. (2010). Oxidant-induced dopamine polymerization for multifunctional coatings. Polym. Chem..

[35] Gao Z.F., Wang X.Y., Gao J.B., Xia F. (2019). Rapid preparation of polydopamine coating as a multifunctional hair dye. RSC Adv..

[36] Muchtar S., Wahab M.Y., Mulyati S., Arahman N., Riza M. (2019). Superior fouling resistant PVDF membrane with enhanced filtration performance fabricated by combined blending and the self-polymerization approach of dopamine. J. Water Process Eng..

[37] Zhu J., Tsehaye M.T., Wang J., Uliana A., Tian M., Yuan S., Li J., Zhang Y., Volodin A., Van der Bruggen B. (2018). A rapid deposition of polydopamine coatings induced by iron (III) chloride/hydrogen peroxide for loose nanofiltration. J. Colloid Interface Sci..

[38] Jiang J., Zhu L., Zhu L., Zhu B., Xu Y. (2011). Surface characteristics of a self-polymerized dopamine coating deposited on hydrophobic polymer films. Langmuir.

[39] Manini P., Panzella L., Napolitano A., d’Ischia M. (2003). A novel hydrogen peroxide-dependent oxidation pathway of dopamine via 6-hydroxydopamine. Tetrahedron.

[40] Liebscher J. (2019). Chemistry of Polydopamine–Scope, Variation, and Limitation. Eur. J. Org. Chem..

[41] Wu H., Liu Y., Mao L., Jiang C., Ang J., Lu X. (2017). Doping polysulfone ultrafiltration membrane with TiO2-PDA nanohybrid for simultaneous self-cleaning and self-protection. J. Membr. Sci..

[42] Smolders K., Franken A. (1989). Terminology for membrane distillation. Desalination.

[43] Kotsilkova R., Borovanska I., Todorov P., Ivanov E., Menseidov D., Chakraborty S., Bhattacharjee C. (2018). Tensile and surface mechanical properties of polyethersulphone (pes) and polyvinylidene fluoride (PVDF) membranes. J. Theor. Appl. Mech..

[44] Howe K.J., Clark M.M. (2002). Fouling of microfiltration and ultrafiltration membranes by natural waters. Environ. Sci. Technol..

[45] Kumar R., Ismail A. (2015). Fouling control on microfiltration/ultrafiltration membranes: Effects of morphology, hydrophilicity, and charge. J. Appl. Polym. Sci..

[46] Tan Y.Z., Mao Z., Zhang Y., Tan W.S., Chong T.H., Wu B., Chew J.W. (2019). Enhancing fouling mitigation of submerged flat-sheet membranes by vibrating 3D-spacers. Sep. Purif. Technol..

[47] Sun W., Liu J., Chu H., Dong B. (2013). Pretreatment and membrane hydrophilic modification to reduce membrane fouling. Membranes.

